# Effects of Different Interventions Aimed at Reducing Dermal and Internal Polycyclic Aromatic Hydrocarbon Exposure Among Firefighters

**DOI:** 10.3390/jox15050150

**Published:** 2025-09-16

**Authors:** Anne Thoustrup Saber, Marie Frederiksen, Simon Pelle Jensen, Vivi Kofoed-Sørensen, Per Axel Clausen, Anja Julie Huusom, Tanja Carøe, Niels Ebbehøj, Maria Helena Guerra Andersen, Ulla Vogel

**Affiliations:** 1 National Research Centre for the Working Environment, 105 Lersø Parkallé, 2100 Copenhagen Ø, Denmark; 2 Department of Occupational and Environmental Medicine, Copenhagen University Hospital, Bispebjerg and Frederiksberg, 23F Bispebjerg Bakke, 2400 Copenhagen NV, Denmark; 3 Department of Occupational and Social Medicine, Holbæk University Hospital, 4B Gl. Ringstedvej, 4300 Holbæk, Denmark; 4 National Food Institute, Technical University of Denmark, Building 202, Henrik Dams Allé, 2800 Kgs. Lyngby, Denmark

**Keywords:** firefighter, polycyclic aromatic hydrocarbon, polycyclic aromatic hydrocarbon metabolite, skin wipe, urine, biomonitoring, intervention

## Abstract

Firefighters are inherently exposed to soot and polycyclic aromatic hydrocarbons (PAHs) at work. In this repeated measures study, we assessed if three different interventions reduced PAH exposure. For each sub-study, the firefighters participated in two sampling periods and thereby served as their own controls. The first period served as baseline, while the second period was the intervention period where the participants received education on health effects of soot, information on own PAH exposure, and participated in one of three interventions: (1) sauna after fire calls, (2) use of fire suits with improved barrier, and (3) showering after every fire call. We recruited 26 firefighters from three different fire stations. Dermal wipes were assessed for 16 PAHs and spot urine for eight hydroxylated metabolites. Pre-shift PAH burden was significantly reduced compared to our previous biomonitoring study. Post-shift levels of two PAH metabolites (1-hydroxypyrene and 1-hydroxyfluorene) were increased for firefighters after a work shift without fire calls compared to pre-shift. The sauna intervention significantly reduced the levels of all the measured urinary PAH metabolites while the dermal PAH exposure remained unaffected. The fire suit intervention yielded more inconsistent results. While standard shower reduced dermal PAH levels, no additional effects were observed for the shower intervention.

## 1. Introduction

Occupational firefighting was recently classified as carcinogenic to humans (IARC group 1) [1]. The occupational exposure of firefighters is very complex and includes exposure to different groups of toxicants. One of these are polycyclic aromatic hydrocarbons (PAHs) of which several are known or suspected human carcinogens [1]. PAHs can be absorbed by inhalation, skin contact, and ingestion [2]. Following absorption, PAH may accumulate in adipose tissue and be gradually released over time. PAHs are metabolized and excreted in the urine as hydroxylated PAHs (OH-PAHs) [1,2]. Several studies on firefighters have indicated that dermal exposure to PAHs is an important exposure route [3,4,5]. Firefighter exposures to PAHs may occur at the fire scene and continue through dermal contact with contaminated gear and clothes carried into vehicles and fire stations [6,7]. This ongoing exposure highlights the importance of knowledge about the exposure and strategies to reduce it in order to minimize the health risks.

Our previous study on recruits during firefighting training showed increased levels of dermal exposure to PAH, which were strongly correlated with urinary excretion of the PAH metabolite 1-hydroxypyrene (1-OHPYR) and DNA damage in blood cells [8]. Furthermore, in the same study, we showed that personal respiratory protection equipment protected the firefighters from pulmonary exposure to particles [8,9]. The results suggested that dermal exposure to PAHs is an important exposure route and that dermal exposure likely contributed to the observed genotoxic effects.

Mitigation of hazard is the most effective exposure control strategy [10], which would include avoiding or reducing the dermal PAH exposure in the first place. Examples of efforts that might be used to avoid or reduce dermal exposure are cleaning of used firefighter gear, separate designated areas for clean and contaminated equipment, and use of inner gloves or suits and hoods made of textile impermeable to particles [5,11,12,13]. Secondly, if the dermal exposure has occurred, mitigation strategies might include cleaning opportunities on scene [14,15] or showering as soon as possible after returning from the fire scene [16]. But a previous study on sauna use after firefighting has only shown a non-significant reduction in urinary OH-PAH metabolite levels [17]. Overall, the knowledge of the effect of different PAH exposure mitigation strategies is limited.

Increased awareness among the firefighters of the importance of reducing the time that PAHs are in contact with the skin to avoid harmful health effects is of course also very important. Feedback on individual exposure has been shown to work as an effective intervention in other occupational fields. For example, a study on farmers showed that personal exposure to inhalable organic dust was reduced by 20–30% following feedback on own exposure combined with instructions on basic measures of prevention [18].

The aim of the present study was to investigate whether PAH exposure could be reduced by different interventions. In collaboration with the project’s reference group and three Danish fire stations, we chose to investigate the three interventions consisting of either sauna, fire suits and hoods with improved particle barrier, and showering after all fire calls. All of these were in combination with education on adverse health effects of soot and feedback on own exposure levels.

## 2. Materials and Methods

### 2.1. Subjects

We recruited firefighters from three Danish fire stations in June 2021 (Station 1, ‘Sauna intervention’), April 2022 (Station 2, ‘Fire suit intervention’), and March 2022 (Station 3, ‘Shower intervention’). Inclusion criteria were firefighters allocated to act as the first firefighters on the fire scene and preferably being non-smokers. The subjects were recruited following oral and written information about the study and signed a written informed consent before entering the study. The firefighters had 24 h work shifts separated by 3 days off (from 8 A.M. to 8 A.M. on the following day).

Prior to the study, the Danish Committee on Health Research Ethics of the Capital Region evaluated the study (H-20034853) and assessed it as a ‘behavioral and method evaluation study’, which is not subject to decision notification (4 November 2020).

### 2.2. Study Design

**Figure 1:**
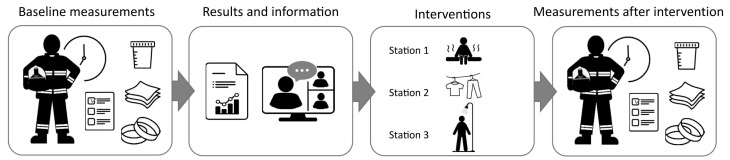
Graphical illustration of the study design.

The design was a repeated measures study, where the firefighters participated in two sampling periods and thereby served as their own controls (Figure 1). The first sampling period served as baseline showing the typical exposure at the fire stations in question. The second sampling period included one of the following interventions: (1) sauna after fire calls, (2) use of fire suits and hoods made of textiles with improved particle barrier, and (3) extra focus on showering after every fire call, which included showering also after minor fires. For each period, samples were collected on days when the participants were on-duty and sampling period covered 1 ½ to 2 months. The timing of the start of the sampling in the intervention periods was decided in collaboration with the participating fire stations. The intervention period occurred approximately 13, 5, and 8 months after the end of the baseline sampling for fire stations 1, 2, and 3, respectively. Before the start of the intervention period, the participants received written information about their individual dermal PAH level and the mean levels at their own stations. This was followed by an invitation to participate in an online meeting where the mean data for the specific fire station was presented. At the online meeting, the participants also received information about the harmful effects of dermal PAH exposure and the specific intervention to be implemented at their fire station during the second sampling period.

### 2.3. Interventions

Before onset of the study, the participating fire stations had decided on the actual interventions in collaboration with the research team. All of these were combined with information on exposure levels and education on effects of PAH exposure. For all three sub-studies, the firefighters showered after fire calls as usual practice during the baseline. This was also the case for the intervention at Fire Station 2. However, at Fire Stations 1 and 3, the showering during intervention was performed following specific instructions as described below.

Sauna (Fire Station 1): During the baseline period, no sauna was used. During the second sampling period, the intervention consisted of using sauna after fire calls. When returning to the fire station after fire calls, the participants were instructed to shower followed by resting in sauna (15 min at 80 °C) (referred to as ‘sauna’ intervention). The participants were instructed to refrain from using the sauna during the baseline sampling to allow for a valid comparison of its effects.

Fire suits (Fire Station 2): Conventional fire suits and hoods were used at fire calls during the baseline measurements. Before the second sampling period, the participants were equipped with new fire suits and hoods with an improved particle barrier. The suits were also designed with extra protection at the waist and with wrist and ankle cuffs to reduce particle entry.

Shower (Fire Station 3): During the baseline period, showering after fire calls was performed as usual practice which meant that the firefighters did not always shower after smaller fires. During the second sampling period, the participants were instructed to shower after every fire call regardless of the type and size of the fire.

### 2.4. Sample Collection and Questionnaire

All samples except wipes of equipment were collected by the firefighters themselves. At an information meeting a few weeks before the first sampling period, the firefighters were instructed how to perform the sampling (Figure 1 and Supplementary Figure S1).

#### 2.4.1. Urine

Internal PAH exposure was quantified by PAH metabolites in urine. The participants were instructed to collect spot urine samples in the morning at the start (referred to as ‘pre-shift’) and the end of the work shift (referred to as ‘post-shift’). The samples were stored at −18 °C at the fire stations, transported to the laboratory in cooling boxes, and stored at −18 °C until analysis.

#### 2.4.2. Skin Wipes

Skin wipes were used to quantify dermal PAH exposure. Silicone wristbands were also collected but these will be reported elsewhere. For both baseline and intervention measurements, the participants were instructed to use skin wipes in the morning at the start of the work shift (referred to as ‘pre-shift’) and the following morning at the end of the work shift (referred to as ‘post-shift’). The ‘pre-shift’ sample was used to characterize baseline exposure after three consecutive days off, whereas the ‘post-shift’ sample quantified exposure following a work shift at the fire station. In the case of a fire call, the participants were instructed to collect additional skin wipes immediately after returning to the station both before (referred to as ‘before shower’) and after showering (referred to as ‘after shower’. The ‘before shower’ sample was used to characterize the dermal exposure after a fire call, whereas the ‘after shower’ sample quantified the effect of showering. For the sauna intervention, the ‘after shower’ sample quantified the effect of shower followed by sauna. During a work shift, up to four different sampling sites of the neck were wiped as shown in Supplementary Figure S1. A cardboard frame defined an area of 18 cm^2^ that was wiped with an isopropanol-soaked cloth (70% isopropanol/water, Mediq Denmark A/S). The procedure was performed as previously described [8] with the deviation of using the cardboard frame, and the sampling was conducted by the participants themselves (as self-sampling or by a colleague). In brief, the participants were instructed to use nitrile gloves and wipe the specific skin area twice with the same wipe, first using one side of the wipe and then the other. The participants were instructed to place the wipes in provided glass vials and store them at −18 °C at the fire station until transport to the laboratory, for continued storage at −18 °C at the laboratory until analysis.

#### 2.4.3. Wipes of Equipment

To assess the efficacy of the cleaning procedure for used gear, we measured the PAHs on cleaned equipment at one of the participating fire stations (Station 3). The measurements were performed at a visit on the fire station after the end of the intervention measurements. Duplicate sets of cleaned equipment were wiped by research personnel to assess residual PAH contamination, using the same type and size of wipes as those used for dermal sampling. The wiped equipment included breathing regulator (mouth piece and purge button), smoke diving mask, inner mask, air cylinder cover, back shield, helmet (liner and shell), and boots. Due to the irregular shapes of the equipment, it was not possible to estimate the wiped area. However, areas of similar size were wiped for duplicates. The results were used an indicative assessment of whether the cleaning procedure removed all PAH from the equipment.

#### 2.4.4. Questionnaire

Participants were asked to fill in a questionnaire during and at the end of each work shift. The questionnaire included questions related to life-style and other exposure to PAHs in the preceding days.

### 2.5. Analysis of Samples

#### 2.5.1. Urinary PAH Metabolite Analysis

Internal PAH exposure was assessed by PAH metabolites in urine. Levels of eight urinary metabolites, i.e., 1-hydroxynaphthalene (1-OHNAP), 2-hydroxynaphthalene (2-OHNAP), 2-hydroxyfluorene (2-OHFLU), 1-hydroxyphenanthrene (1-OHPHE), 2 and 3-hydroxyphenanthrene (2 + 3-OHPHE), 4-hydroxyphenanthrene (4-OHPHE), and 1-OHPYR were measured as previously described [19] with some modifications but unchanged Limit of Quantification (LOQ). In brief, urine was thawed and homogenized; 800 µL urine were transferred to centrifuge tubes and 100 µL ^13^C-labeled internal standard solution (‘CDC OH-PAH Spiking Standard’, Cambridge Isotopes Laboratories, Inc. (Tewksbury, MA, USA)diluted 1000 times in methanol) and 100 µL β-glucuronidase/buffer solution were added. Samples were incubated for 16–20 h at 37 °C and centrifuged; the supernatant was analyzed immediately or stored at −20 °C until analysis. The analysis was performed by online SPE-LC-MS/MS with an injection volume of 100 µL, the SPE column was an Agilent Zorbax SB-C18 (2.1 × 30 mm, 3.5 µm) cartridge column, and the analytical column a Phenomenex Synergi Hydro RP C18 (2 × 100 mm, 2.5 µm, 100 Å). The modifications included the samples being run on a new LC-MS (Agilent Infinity II 1290 LC and 6495C triple quadrupole MS, Agilent Technologies, Santa Clara, CA, USA) and adding back-flushing of the SPE column after each run; the full details of the system and the program can be found in the Supplementary Materials (Supplementary Tables S1 and S2 and Figure S2). It was not possible to separate 2 and 3-OHPHE so they were reported as the sum. For all urine samples, the concentration of urinary creatinine was used to standardize for diuresis in all urine concentrations measured by colorimetry on a PentraC400 (Horiba ABX SAS, Grabels, France) as described previously [19].

#### 2.5.2. PAHs in Skin and Equipment Wipes

The PAHs were extracted from the wipes using 5 mL cyclohexane by sonication for 60 min (Branson 5200, Branson Ultrasonics Corporation, Brookfield, CT, USA). Afterwards, 0.9 mL of the supernatant was transferred to a glass vial and added 100 µL of deuterated internal standard (100 ng/mL); the samples were stored at − 18 °C until analysis. The extracts were analyzed by gas chromatography with tandem mass spectrometry (GC–MS/MS) using a Bruker SCION TQ (Bruker Daltonics, Bremen, Germany). The analysis was performed by injection of 1 µL of sample extract to a programmable temperature vaporizing inlet (PTV) in splitless mode at 280 °C connected to a 60 m column (SGE HT8, i.d.: 0.32 mm; film thickness: 0.25 μm) with 1 mL/min helium flow. The GC oven program started at 80 °C (hold 1 min), increased to 280 °C at 10 °C/min (hold 20 min), and then increased to 310 at 20 °C/min (hold 15 min) [20].

In total, 16 PAHs were quantified by GC-MS/MS in multiple reaction monitoring (MRM) mode; the full list of analytes along with retention time, collision energy, and transitions are provided in Supplementary Table S3. Complete separation was not possible for benzo[b]fluoranthene and benzo[k]fluoranthene, and therefore they were reported as the sum (benzo[k+b]fluoranthene). The LOQ was set to 10 times signal-to-noise ratio and typically ranged from 0.5 to 4.3 ng/mL depending on the PAH. LOQ was calculated as ng/cm^2^ for wipes (with a wipe area of 18 cm^2^) and as ng/sample for wipe samples from equipment (with an extraction volume of 5 mL).

All samples were blank corrected by subtracting the mean of lab blanks; levels below LOQ were set to zero in the summary data. Due to instable and occasionally high blank levels, the levels of dibenz(ah)anthrancene, ideno(123cd)pyrene, and benzo(ghi)perylene should be interpreted with caution and therefore they were not included in the statistical analysis.

### 2.6. Statistical Analysis

Urinary metabolite data were analyzed with a linear mixed effect model using R version 4.4.1 and the lme4 package [21]. The data were log transformed due to a skewed residual distribution and the few non-detects (3.0% for 1-OHPYR, 0% for ∑OHNAP, 2-OHFLU, and ∑OHPHE) were imputed by half of the LOQ. The statistical testing of the urinary metabolite data included two different approaches: a simple model (referred to as Model 1) and a more comprehensive model (referred to as Model 2). In Model 1, all three stations were included in the model and the following assumptions were made: no differences in the levels of pre-shift samples between the baseline and intervention periods, no variation in the effect of fire extinguishing across the three fire stations, and no differences in the levels in post-shift samples without fire extinguishing between the baseline and intervention periods.

Study subjects were included as a random factor; multiple fixed effects were included in the model: Shift (2 levels—Pre/post), Fire call (2 levels—Yes/No, indicating firefighting activities during the shift), and Intervention (4 levels—No/Sauna/Fire suit/Shower, indicating intervention type). We also controlled for external PAH exposure (smoking, exposure to other sources of smoke the past 3 days, part-time job with smoke exposure, use of chewing tobacco, use of creams with PAHs, intake of grilled and smoked food the past three days) before work shifts (information retained from questionnaire, 2 levels—yes/no), which had no significant effect on the final estimates. Effects are reported as percentage changes, calculated using the β estimates from the log-transformed model with the following equation: ((e^Estimate^) − 1) × 100.

Model 1: A mixed model was applied using the following formula:

(1) $$Y={\beta_{0}+\beta }_{1}X_{1}+\beta_{2}X_{2}+\beta_{3}X_{4}+\beta_{4}X_{4}+\gamma +\varepsilon ,$$

where *β_0_* represents the baseline level before the shift, *X_1_* to *X_4_* represent the fixed-effect predictors (Shift, Firefighting activity during shift, different intervention strategies—Sauna, Fire suit, and Shower) and PAH exposure) and *β_1_* to *β_4_* the coefficients of effect of each predictor on the response variable *Y*, assuming all other variables are held constant, γ is the random effect associated with individual differences (person), and *ε* the residual error term.

$$\beta_{0}=Pre-shift\left(Baseline\;level\right),\;\beta_{1}=Shift\left(Levels:Pre,Post\right),\;\beta_{2}=Firecall\left(Levels:No,Yes\right),\;\beta_{3}=Intervention\;(Levels:No,\;Sauna,\;Fire\;suit,\;Shower),\;\beta_{4}=\mathrm{Selfreported}\;\mathrm{PAH}\;\mathrm{exposure}\;(Levels:No,Yes),\;\gamma =\mathrm{Random}\;\mathrm{effect}\left(Person\right)\;\varepsilon =Error$$

In Model 2, we included terms to allow for differences in the levels of pre-shift samples between the baseline and intervention periods and differences in the levels of post-shift samples (without fire extinguishing) between baseline and intervention periods (labelled ‘Awareness’). Additionally, each station was modeled separately, as the baseline levels, post-shift levels, and effect of fire call were not assumed to be the same. A mixed-effect model was applied using the same formula as above, with the addition of two terms: $\beta_{5}=\mathrm{Intervention}\;\mathrm{period}\left(Levels:No,Yes\right)$ and $\beta_{6}=Awareness\;(Levels:No,Yes)$.

Wipe data was analyzed similar to the urinary data except for the effect ‘Shift’ (3 levels—Pre-shift, Before shower, After shower/Post-shift) and the effect ‘Self-reported PAH exposure’ was excluded due to limited data on the subset analyzed. Due to instable and occasionally high blank levels, the levels of dibenz(ah)anthrancene, ideno(123cd)pyrene, and benzo(ghi)perylene were not included in the statistics. The non-detects were imputed by half of the lowest detected value.

Wipe data was analyzed with an additional model to test the effect of ‘standard’ showering.

Model 3: A mixed model was applied using the following formula:

(2) $$Y={\beta_{0}+\beta }_{1}X_{1}+\beta_{2}X_{2}+\gamma +\varepsilon$$

where

$$\beta_{1}=Shower\;(Levels:Before,\;After)\;\beta_{2}=Intervention\;(Levels:No,\;Yes)$$

In Model 3, we included baseline wipe samples before and after shower from all three fire stations, as well as before and after shower wipe samples from Station 3 (shower intervention) from the intervention period.

## 3. Results

In total, we recruited 26 firefighters in the study including 11 at Fire Station 1, 8 at Fire Station 2 and 7 at Fire Station 3. A description of the demographic information of the participants is found in Table 1. All the participants were male and the mean age was 44.9 years at baseline. The mean body mass index (BMI) was 27.0 kg/m^2^ based on self-reported height and weight. No subjects had a BMI below 18.5, 9 subjects had a BMI between 18.5 and 24.9, 13 subjects between 25.0 and 29.9, and 4 subjects 30 or above. Nineteen of the subjects were never smokers, six were previous smokers, and one was a current smoker.

### 3.1. Sample Collection

The planned experimental setup was to collect samples for one month for each exposure period (baseline and intervention) corresponding to eight work shifts per participant, and expecting three work shifts with fire calls per firefighter based on information from the participating fire stations. However, all fire stations had fewer fire calls than expected and therefore the sample collection periods were extended to last for 1.5–2 months aiming at 12–16 work shifts per participant for each exposure period. However, even after the extended sampling period, not all of the recruited firefighters had been on fire calls during the sampling period (Table 2).

The extended sample collection period primarily resulted in an increased number of samples without fire calls. Looking at the urine samples collected post-shift at baseline and with intervention combined, 75%, 56%, and 89% were from shifts without fire calls for stations 1, 2, and 3, respectively. All urine samples were analyzed regardless of exposure status. Due to budget limitations, only a subset of the dermal wipe samples were analyzed prioritizing those collected after fire calls and one or two matching samples from shifts with no fire calls. The numbers of analyzed dermal wipes are provided in Table 2.

### 3.2. Urinary PAH Metabolites

The mean and median urinary PAH metabolite levels in firefighters’ pre-shift samples, combined for both sampling periods, are shown in Table 3. The median (P5, P95) of the total urinary OH-PAHs was 2.13 (1.05; 7.85) µmol/mol creatinine where the hydroxy naphthalenes constituted the majority (85%) of the measured OH-PAHs. The median (P5, P95) urinary 1-OHPYR was 0.022 (0.006; 0.10) µmol/mol creatinine.

**Figure 2:**
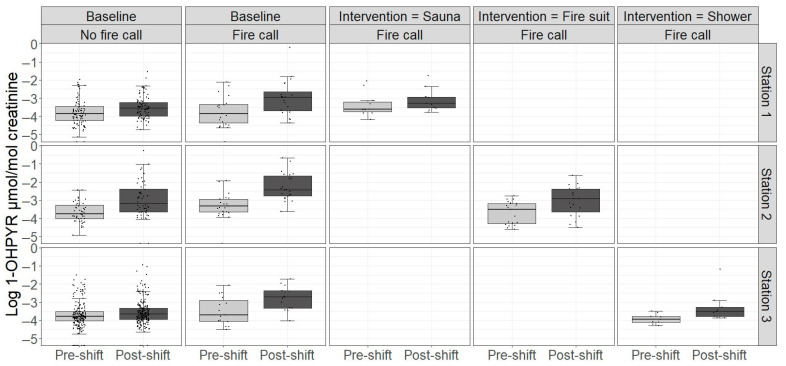
Urinary levels of creatinine-adjusted 1-hydroxypyrene (1-OHPYR) in participants at baseline and in the intervention period across work shifts with and without fire calls. Boxplots represent the median and interquartile range; whiskers extend to 1.5 times the interquartile range. Points represent individual data points.

The urinary levels of OH-PYR are shown in Figure 2 while the rest can be found in Supplementary Figure S3a–c and in Supplementary Table S4. Table 4 and Table 5 show the percent change in urinary levels of PAH metabolites using statistical Models 1 and 2, respectively. In the simple Model 1, being on-duty at the fire stations on a work shift with no fire calls increased the urinary levels of 2-OHFLU by 8.3% (95% CI: 2.0, 15.0) and 1-OHPYR by 37.7% (95% CI: 24.6, 52.2) (Table 4).

The effects of the three different interventions on urinary PAH metabolites were estimated using two different statistical models as described in Materials and Methods.

The sauna intervention significantly reduced urinary OHFLU, ∑OHPHE, and OHPYR in both models with similar reductions (28.1%, 33.6%, and 36.9% in Model 1, and 33.0%, 35.0%, and 37.5% in Model 2, respectively). The ∑OHNAP level was significantly reduced in Model 2 (41.7%) but only non-significantly reduced in Model 1. In the fire suit intervention, the only significant reduction was observed for OHPYR in Model 1 (41.1%) while a non-significant increase in ∑OHNAP was observed in Model 2. There was no effect of the shower intervention on any urinary PAH metabolites in either model. However, in Station 3, who tested showers in the intervention, post-shift levels of OHPYR without fire calls were significantly lowered in the intervention period (Table 5).

There was no clear effect of the ‘awareness’ defined as the change in post-shift levels without fire calls between the baseline and intervention periods as most PAH metabolites changed non-significantly in both directions. The only exceptions were OHPYR which was significantly reduced at Station 3 and OHFLU which was significantly increased at Station 1.

To assess the effect of the inclusion of a single current smoker in the study, we have analyzed the 1-OHPYR data omitting the smoker and in another analysis adjusting for smoking. Omitting the smoker reduces the power of the analysis due to the limited number of participants, while controlling for smoking did not affect the results. 

### 3.3. PAH from Skin Wipes

**Figure 3:**
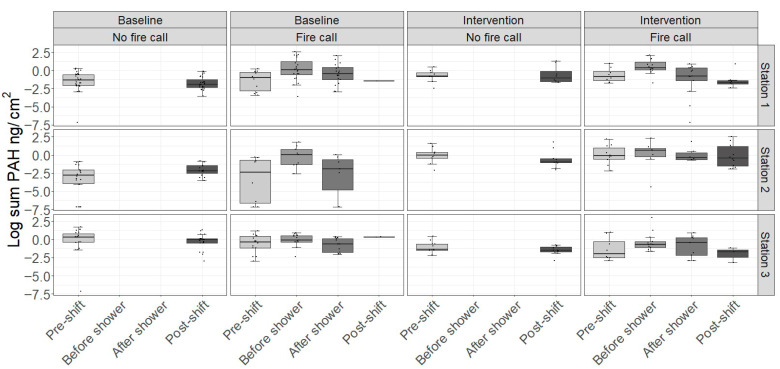
Dermal levels of the sum of the concentrations of 13 PAHs* in participants at baseline and in the intervention period (Station 1: Sauna, Station 2: Fire suit, Station 3: Shower) across work shifts with and without fire calls. Boxplots represent the median and interquartile range; whiskers extend to 1.5 times the interquartile range. Points represent individual data points. * In total, 16 PAHs were quantified. However, due to instable and occasionally high blank levels, the levels of three PAHs (dibenz(ah)anthrancene, ideno(123cd)pyrene, and benzo(ghi)perylene) were neither reported nor included in the statistics.

The mean and median levels of dermal ∑PAH on the neck of firefighters pre-shift for both sampling periods combined are shown in Table 3. The median (P5, P95) of ∑_13_PAH was 0.42 (0.001; 3.42) ng/cm^2^ for the 13 reported PAHs. The sum of the 16 analyzed PAHs (∑_16_PAH) is also provided in Table 3, but should be used with caution and only for comparison with earlier studies. The dermal ∑_13_PAH levels are shown in Figure 3, while Table 6 and Table 7 show the percentage change in dermal ∑_13_PAH using statistical Model 1 and 2, respectively. The levels of the individual PAHs are shown in Supplementary Table S5a–c.

Compared to pre-shift, the level of ∑_13_PAHs on the skin increased by 232% before showering after a fire call (Model 1, Table 6). The ∑_13_PAH was increased 75% following showering after a fire call compared to post-shift levels without fire calls (Model 1, Table 6).

Both Model 1 and 2 showed that the sauna intervention reduced the dermal ∑PAH non-significantly. The fire suit intervention showed a non-significant increase in Model 1 (Table 6), while a statistically significant reduction was seen using Model 2 (Table 7).

There was no effect of the shower intervention in either model. However, Model 3 showed that the ‘standard shower’ as measured as the difference in dermal PAH before and after shower in the baseline period significantly reduced the dermal level of PAHs by −63.9 (95% CI: −78.3; −39.5) (Table 8).

There was no effect of the ‘awareness’ defined as the change in post-shift levels between the baseline and intervention periods without a fire call.

Overall, although they are not significant, the skin wipes show similar trends as the PAH metabolites.

In summary, the sauna intervention reduced the urinary excretion of most of the PAH metabolites in both statistical models, whereas non-significant reductions of dermal PAH were seen. Results regarding the fire suit intervention were somewhat more inconsistent. Only urinary OH-PYR was significantly reduced in the simple statistical model, whereas none were significantly reduced in Model 2. For dermal PAH, the two statistical models yielded diverging results. For the shower intervention, no significant effects were seen.

### 3.4. PAH on Equipment

**Figure 4:**
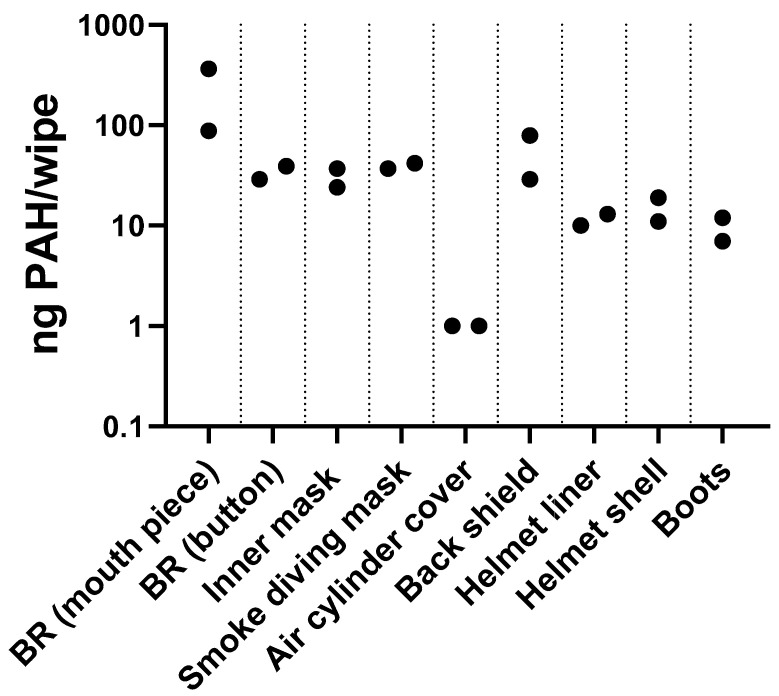
PAH levels on firefighter equipment after usual decontamination procedures at Fire Station 3. Points represent individual results. BR: breathing regulator; button: purge button.

PAHs were detected in wipe samples collected from cleaned and ready-to-use equipment from Station 3 (Figure 4). All samples had detectable PAH levels, suggesting that cleaned equipment could contribute to PAH exposure.

## 4. Discussion

The aim of this study was to assess the effects of three different interventions aiming at reducing PAH exposure during firefighting. All interventions consisted of a combination of education on adverse health effects of PAH exposure, personal feedback on own dermal PAH exposure levels, and an intervention that was selected in collaboration with the fire stations. Importantly, the study showed that being on-duty with no fire extinguishing resulted in higher levels of 2-OHFLU (8.2% increase) and 1-OHPYR (37.7% increase) compared to pre-shift. The study also showed that internal exposure to PAHs assessed by urinary PAH metabolite levels and dermal PAH exposure was higher for firefighters who were on fire calls during duty. The sauna intervention significantly reduced the levels of 2-OHFLU, ∑OHPHE, and 1-OHPYR, with the largest reduction in 1-OHPYR (37%), while the decrease in ∑OHNAP was only significant in one of the statistical models. The fire suit intervention also numerically reduced the levels of all measured PAH metabolites. However, only the decrease in 1-OH-PYR levels was statistically significant with a 41% reduction and only in the simple statistical model. The fire suit intervention numerically increased dermal PAH in the simple model but statistically decreased dermal PAH in the more comprehensive model. No significant effects were seen for the shower intervention. However, the effect of showering assessed in the baseline period by comparing dermal PAH before and after showering show that ‘standard showering’ reduced the dermal PAH level.

Urinary levels of OHPYR both pre- and post-shift at baseline and intervention were much lower than observed in our first biomonitoring study of firefighters (Biobrand 1), which was performed in 2016 at Station 1 [22]. In Biobrand 1, the urinary mean levels of 1-OHPYR were 0.52 µmol/mol creatinine (SD: 0.51) before shift and 0.6 µmol/mol creatinine (SD: 0.56) after shift. In the present study, the mean levels of 1-OHPYR during the baseline period at the same fire station were 0.03 µmol/mol creatinine (SD: 0.03) pre-shift and 0.1 µmol/mol creatinine (SD: 0.2) post-shift with fire calls in samples collected 5–7 years later. The 1-OHPYR measurements from the Biobrand 1 and Biobrand 2 studies are not directly comparable as the analytical method for the 1-OHPYR differ, with the one used in Biobrand 2 being more specific than the previous. Nevertheless, considering the magnitude of the difference in urinary 1-OHPYR levels between the two studies, this suggests a substantial reduction in the internal PAH exposure over time. Similarly, in Biobrand 1, we reported dermal mean pre-shift values of 232.5 µg/m^2^ (SD: 78.4) corresponding to 23.3 ng/cm^2^ (SD: 7.8) [22]. These are substantially higher than in the current study where we reported 2.61 ng/cm^2^ (SD: 4.57). The reduced baseline levels in the present study are likely a consequence of a number of initiatives to reduce dermal PAH exposure at the participating fire stations that were implemented following our first study. The nature of the soot-reducing initiatives implemented between these two studies differed between the fire stations. Examples of these initiatives include using special bags for contaminated equipment that open during wash in the washing machine and separate designated areas for clean and contaminated equipment, and specially designed machines for cleaning hoses without any human contact—just to mention some. We believe that the dialogue with the fire stations and other stakeholders regarding the outcome of our previous study highlighting the importance of dermal PAH exposure for genotoxic effects has contributed to the increased focus on hygiene. In addition, the Danish Working Environment Authority launched a campaign in 2019 aimed at getting firefighters to shower immediately after fire calls [23]. The campaign directly referred to the Biobrand 1 project.

In the present study, we observed a 3-fold difference in mean 1-OHP levels between pre-shift (0.03 µmol/mol creatinine) and post-shift (0.1 µmol/mol creatinine) samples collected after a shift with a fire call. Post-fire levels among the participating Danish firefighters are similar to the levels in a non-occupationally exposed German background population of 0.30 µg/g creatinine (equals 0.16 µmol/mol creatinine) for non-smokers and well below 0.73 µg/g creatinine (equals 0.38 µmol/mol creatinine), the level observed for smokers [24]. The observed post-shift 1-OHP average concentration of 0.1 µmol/mol creatinine is also below the proposed no-observed genotoxic effect level of 1.0 µmol/mol creatinine [25]. When considering the different types of fires and the different timing of the collection of urine samples post-fire, the pre-shift and after-fire levels as well as the difference between pre- and post-shift is quite similar to what has been seen in other recent firefighter studies: In a German study of 77 firefighters, the mean levels of 1-OHPYR were doubled when measured 2–4 h after fire extinguishing (0.27 µg/g creatinine (equals 0.14 µmol/mol creatinine)) compared to baseline (0.13 µg/g creatinine (equals 0.07 µmol/mol creatinine)) [24]. Similarly, an American study of 141 firefighters observed a 1.6–2.2 fold increase in 1-OHPYR levels 2–4 h after fire extinguishing [26], while a Canadian study of 22 firefighters reported 1.5 fold change in 1-OHPYR levels from a geometric mean of 0.04 µg/g creatinine (equals 0.02 µmol/mol creatinine) pre-fire to 0.06 µg/g creatinine (equals 0.03 µmol/mol creatinine) in pooled post-fire samples collected 18 h after firefighting [14].

In the present study, the majority of the firefighters’ work shifts were without fire calls (Table 2). Even though firefighters have other tasks outside the fire station than firefighting such as assistance at car accidents, this means that firefighters spent a substantial part of their working time at the fire station. Work at the fire station included but was not limited to cleaning of used equipment and maintenance of smoke diving equipment and automobiles. Therefore, in addition to the exposure during actual firefighting, the secondary exposure at the fire station may be important for the overall occupational exposure for firefighters. Nevertheless, most studies on firefighters have focused on the exposure during actual fire extinguishing and only a few have assessed exposure at fire stations. The results from our study showed that a 24 h work shift without fire calls was associated with a substantial increase in internal exposure to PAHs measured as urinary PAH metabolites (the 1-OHPYR and 2-OH-FLU levels were increased by 38% and 8%, respectively). Two recent studies used silicone wristbands as passive samplers to assess the PAH exposure of firefighters when off-duty and on-duty with and without fire calls [27] and in different locations at the fire stations [28]. The study by Lavasseur and colleagues showed similarly to the present study that staying at the fire station without responding to fire calls increased the exposure to PAHs. In that study, specific PAHs (phenanthrene, pyrene, benzo(j)fluranthene, benzo(k)fluoranthene, indeno(123-cd)pyrene, and benzo(ghi)perylene) increased significantly when on-duty without a fire event as compared to off-duty [27]. In the study by Papas and colleagues, wristbands were placed for one month in different locations at a fire station: vehicle bay, sleeping quarters, living room area, and turnout (bunker) gear storage room. In addition, wristbands were placed in administrative office areas adjacent to the fire station (linked by a corridor with doors at each end). The results showed that while office areas had significantly lower PAHs than fire station areas, the living room areas at the fire station, vehicle bays, and truck cabins had comparable (and higher) levels of high molecular weight PAHs [28].

To assess the efficacy of the cleaning procedure for used gear, we measured the PAHs on cleaned equipment at one of the participating fire stations (Station 3). Although the number of samples collected in the present study was low, the results showed that cleaning of equipment did not always remove all of the PAHs. This may result in dermal exposure both during the cleaning process and when handling the cleaned equipment. Previous studies have shown that the dose of detergent is important when cleaning textiles used for firefighting gear: The cleaning efficacy of PAHs was increased more than 90% when higher concentrations of detergent than recommended were used [12]. Furthermore, presoaking has been shown to result in greater removal of high molecular weight PAHs [13]. A recent review on air pollution inside fire stations identified firefighting equipment and personal protective equipment as some of the main sources of pollution at fire stations [29]. The review also highlighted that more knowledge on air concentrations of pollutants in fire stations is needed. The results from the present study and the above-mentioned studies show that despite many ongoing hygiene efforts on fire stations to reduce soot contamination, there is still room for improvement and a need for documenting the effects of these efforts.

The background for the sauna intervention in Station 1 was a common perception among firefighters that sauna use reduces soot exposure and smell. The results from our study showed 37, 34, and 28% decreases in mean urinary 1-OHPYR, ∑OHPHE, and 2-OHFLU, respectively, following sauna treatment (including showering before sauna). ∑OHNAP was non-significantly reduced by 24%. This level of reduction is similar to a study of US firefighters (n = 12), where infrared sauna treatment for 20 min at 49 °C non-significantly reduced the geometric mean of total OH-PAHs measured in 12 h post-exposure urine by 43.5% compared to a control group with no sauna session (n = 12) [17]. The largest reduction, though not statistically significant, was seen for urinary OHNAP metabolites [17]. Still, more studies are needed to be able to evaluate the effect of sauna on the systemic PAH exposure and it should be noted that the sauna intervention most likely was a combination of sauna and an extra shower. In addition, the possible effects of sauna on other health outcomes, including the additional heat and cardiovascular stress, need to be considered. Multiple cardiovascular endpoints (including thrombus formation, vascular function, and myocardial ischemia) were negatively affected in a study of 19 experienced firefighters following 20 min of firefighting training exercises [30]. It would be a concern if using a sauna after firefighting increases cardiovascular strain. We did not collect measurements on the temperature for the participating firefighters. However, in the study by Burgess et al., the core temperature was not increased during or following sauna treatment, although heart rate was elevated after the sauna, suggesting heat stress [17].

The results showed a 41% significant reduction in 1-OHPYR when using clothing with an improved particle barrier in Model 1 but was non-significant in Model 2. At this particular station, the pre-shift levels were quite different between the baseline and intervention period, but in Model 1 these are assumed to be at the same level; therefore, the significant result obtained in Model 1 may be affected by this. The diverging results by using the two models did not allow a definitive conclusion about the impact of the fire suit intervention. In a study on mannequins dressed with different designs (knitted hoods vs. particulate-blocking hoods, turnout jacket with zipper closure vs. hook and dee closure) and exposed to airborne PAHs, particulate-blocking hoods reduced PAHs by approximately 30% compared to knit hoods [31].

Our results show that ‘standard shower’ as used in the baseline period removes dermal PAHs. The intervention in Station 3 aimed to enhance the removal of dermal PAHs after firefighting by implementing showers following all fire calls. However, the focus on showering after all the fire calls did not affect the exposure levels, neither when measured as dermal PAH exposure nor as urinary PAH metabolites. Similar results have been obtained in a recent study on Canadian firefighters [14]. In that study, different on-site attempts to remove PAHs from the skin were tested. Washing using soap and water but not the use of two different types of commercial skin wipes reduced the dermal level of PAHs. However, none of the attempts reduced the internal level of PAHs (measured as urinary PAH metabolites) compared to a control group that did not use any on-site decontamination [14]. In contrast, another Canadian study found that enhanced skin hygiene reduced urinary 1-OHPYR in wildland firefighters when PAHs had been accumulated on the skin (only for firefighters with naphthalene on skin wipes at the end of shift) [32].

The extra focus on hygiene was for all three interventions combined with feedback on dermal PAH exposure before the second exposure scenario was initiated. On shifts with fire calls, the design of our study did not allow us to distinguish the effects of the awareness following feedback and the physical hygiene interventions. However, for shifts without fire calls, a comparison between post-shift levels of dermal PAH and urinary PAH metabolites during intervention and baseline was a proxy for the effect of awareness.

We found no consistent effects of awareness on urinary PAH levels and non-significantly reduced dermal PAH levels. Thus, we were unable to demonstrate the effects of increased awareness.

Feedback to farmers on own exposure to dust has been shown to reduce their dust exposure by 20–30% [18]. In another study, three different behavioral interventions to reduce nickel exposure in an Indonesian nickel processing plant were tested [33]. In the three interventions, the participants received (1) an educational booklet about nickel, potential health effects, and preventive measures, (2) the booklet plus an oral presentation, and (3) booklet, oral presentation, and personal feedback on their levels of nickel in serum and urine. All three different interventions resulted in a significant reduction in serum and urine nickel levels. Since there was no difference across the three intervention groups, it seems that the simplest intervention, namely the booklet, was as effective as the more comprehensive (and more costly) interventions.

This intervention study on real firefighters has a strong design. We performed repeated measures on firefighters in two sampling periods (with several measures with and without intervention), where the firefighters served as their own controls. We evaluated a comprehensive PAH exposure, including dermal and urine samples, assessed for 16 PAHs and eight different PAH metabolites.

The current study also has a number of limitations. The most important limitations are the limited number of measurements from work shifts with fire calls and the limited number of firefighters included in the study. Furthermore, we note that due to the different timing of the fires during the work shift and the time course for the excretion of PAH metabolites [19], the urine samples collected at the end of the work shift captured the excretion at different time points relative to the actual exposure time, which would have had the largest effect on OHNAPs as they have the shortest half-life. In addition, we were not able to control that the firefighting activities differed in length and type, and that other activities with potential PAH exposure were performed at the station while on duty (handling of equipment, vehicles, or others). Another limitation is that wiping of up to four different areas on the neck might have led to some “overlaps” when wiping, increasing the variation in the results. In addition, differences in sampling such as, e.g., self-sampling or sampling by different colleagues, could influence the PAH sample collection. The automated analysis of urinary PAH metabolites allowed us to analyze all available samples, whereas we chose to limit the number of analyzed samples for the more costly analysis of dermal PAH. In addition, skin temperature may affect PAH absorption [34,35]. Core temperature would mostly be a concern for cardiovascular strain and dehydration (that can affect excretion of creatinine). Even though dermal neck exposure has been regarded the most relevant area for wiping in previous studies [8,36], it may not be representative for the whole-body exposure. Regarding the comparison of urinary content in Biobrand 1 and 2, different analytical methods were used that may not be directly comparable: Biobrand 1 samples were analyzed with the less specific fluorescence detector following manual clean-up of a very large volume of urine (40 mL), which makes the method more prone to be affected by the matrix background or other similar compounds. Finally, 1 out of the 26 firefighters reported that he was a current smoker. Omitting the smoker in the statistical analysis reduces the power of the analysis due to the limited number of participants. However, controlling for smoking did not affect the results.

## 5. Conclusions

The present intervention study aiming at reducing exposure to PAHs in firefighters showed that (1) firefighters are exposed to PAH during the work shift in the absence of fire calls, and (2) firefighters on fire calls have further increased PAH exposure during work shifts with fire calls. The sauna intervention significantly reduced the levels of urinary PAH metabolites in post-shift spot urine whereas the effect of the fire suit intervention was less clear. ‘Standard shower’ as measured as the difference in dermal PAH before and after shower in the baseline period significantly reduced the dermal level. However, showering as intervention after every fire call had no significant effect.

## Figures and Tables

**Table 1:** Self-reported demographic information for the study participants (*n* = 26).

		*n* (%)	Mean (SD)
Gender			
	Male	26 (100)	
	Female	0 (0)	
Personal/life-style			
	Age (years)		44.9 (11.5)
	Height (cm)		181.9 (6.3)
	BMI (kg/m^2^)		27.0 (2.9)
	Smoking status		
	Never	19 (73)	
	Previous	6 (23)	
	Current	1(4)	
Other PAH exposure ^1^		18 (69)	

^1^ Other PAH exposure is based on participants’ self-reported exposure to PAHs from other sources than fire-fighting activities (smoking, exposure to other sources of smoke the past three days, part-time job with smoke exposure, use of chewing tobacco, use of creams with PAHs, intake of grilled and smoked food the past three days).

**Table 2:** Number of analyzed urine and skin wipe samples (number of firefighters) stratified by station and sampling time.

Station			1					2					**3**			
Intervention			Sauna					Fire Suit					**Shower**			
Sampling Time ^2^			Pre-Shift ^1^	B. Shower	A. Shower	Post-Shift ^1^		Pre-Shift ^1^	B. Shower	A. Shower	Post-Shift ^1^		Pre-Shift ^1^	B. Shower	A. Shower	Post-Shift ^1^
Urine samples																
	No fire call ^3^		90 (11)			93 (11)		49 (8)			50 (8)		194 (7)			196 (7)
	Baseline w. fire call		20 (7)			20 (7)		20 (8)			20 (8)		15 (5)			15 (5)
	Intervention w. fire call		9 (5)			11 (5)		19 (7)			19 (7)		9 (6)			9 (6)
Skin wipe samples																
	No fire call ^3^		28 (10)			27 (10)		18 (7)			15 (7)		23 (7)			21 (7)
	Baseline w. fire call		10 (6)	20 (7)	18 (7)	1 (1)		8 (5)	9 (5)	7 (4)	0 (0)		14 (5)	14 (5)	13 (5)	2 (2)
	Intervention w. fire call		8 (4)	15 (6)	13 (6)	6 (4)		10 (6)	7 (4)	6 (4)	12 (6)		8 (6)	13 (7)	9 (6)	3 (3)

^1^ The terms ‘Pre-shift’ and ‘Post-shift’ refer to sampling at the beginning and end of the work shift, respectively, ^2^ B: Before; A: After, ^3^ The work shifts without fire calls at the baseline and intervention were combined.

**Table 3:** Median and mean levels of dermal PAH (ng/cm^2^) and urinary PAH metabolites (µmol/mol creatinine) in the subjects pre-shift (baseline and intervention combined).

Dermal PAH		Median (P5, P95)	Mean (SD)	Detection Frequency
	Naphthalene	<LOQ (<LOQ; 0.15)	0.047 (0.2)	23.3%
	Acenapthylene	<LOQ (<LOQ; 0.039)	0.0094 (0.02)	35.8%
	Acenaphtene	<LOQ (<LOQ; 0.35)	0.0066 (0.02)	17.3%
	Fluorene	<LOQ (<LOQ; 0.083)	0.022 (0.07)	33.9%
	Phenanthrene	0.026 (<LOQ; 0.66)	0.13 (0.3)	67.5%
	Anthracene	<LOQ (<LOQ; 0.14)	0.026 (0.07)	33.9%
	Fluoranthene	0.006 (<LOQ; 0.62)	0.11 (0.3)	58.9%
	Pyrene	<LOQ (<LOQ; 0.25)	0.052 (0.1)	49.3%
	Benzo(a)anthracene	<LOQ (<LOQ; 0.35)	0.064 (0.2)	20.9%
	Chrysene	<LOQ (<LOQ; 0.52)	0.1 (0.3)	27.2%
	Benzo(b/k)fluoranthene ^1^	<LOQ (<LOQ; 1.08)	0.21 (0.6)	29.6%
	Benzo(a)pyrene	<LOQ (<LOQ; 0.31)	0.063 (0.3)	13.7%
	∑_13_PAH	0.42 (0.001; 3.42)	0.83 (1.2)	
	∑_16_PAH ^2^	0.70 (0.001; 11.38)	2.61 (4.57)	
Urinary PAH metabolites		Median (P5, P95)	Mean (SD)	
	∑Hydroxy naphthalene	1.8 (0.77; 7.16)	2.71 (3.1)	100%
	2-Hydroxy fluorene	0.085 (0.034; 0.27)	0.12 (0.16)	100%
	∑Hydroxy phenanthrene	0.15 (0.065; 0.61)	0.21 (0.17)	100%
	1-Hydoxry pyrene	0.022 (0.006; 0.10)	0.031 (0.030)	97.0%
	∑OH-PAH ^3^	2.13 (1.05; 7.85)	3.07 (3.2)	

^1^ The sum of benzo(b)fluoranthene og benzo(k)fluoranthene. ^2^ In total, 16 PAHs were quantified, but due to instable and occasionally high blank levels, the levels of three PAHs (dibenz(ah)anthrancene, ideno(123cd)pyrene, and benzo(ghi)perylene) were not included in the statistics and ∑_16_PAH should only be used for the purpose of comparison with previous studies. ^3^ The sum of OH-PAHs.

**Table 4:** Percentage change in urinary excretion levels of PAH metabolites (95% CI) using statistical Model 1 ^1^.

Factor	∑OHNAP	OHFLU	∑OHPHE	OHPYR
Post-shift (w/o fire call) ^2^	−2.0 (−8.6; 5.1)	**8.3 (2.0; 15.0)**	5.1 (−1.0; 11.6)	**37.7 (24.6; 52.2)**
Post-shift (w fire call) ^3^	**39.1 (20.9; 60.0)**	**36.3 (20.9; 53.7)**	**50.7 (32.3; 69.9)**	**99.4 (64.9; 143.5)**
Intervention (Sauna) ^4^	−23.7 (−45.1; 5.1)	**−28.1 (−45.1; −4.9)**	**−33.6 (−49.8; −12.2)**	**−36.9 (−59.7; −1.0)**
Intervention (Fire suit) ^4^	−11.3 (−31.6; 15.0)	−14.8 (−31.6; 6.2)	−11.3 (−29.5; 9.5)	**−41.1 (−58.9; −15.6)**
Intervention (Shower) ^4^	4.1 (−26.7; 46.2)	20.9 (−9.5; 61.6)	1.0 (−25.2; 35.0)	−18.9 (−49.3; 31.0)

Numbers in bold indicate statistical significance. ^1^ In Model 1, the assumptions were no differences in the levels of pre-shift samples between the baseline and intervention periods, no variation in the effect of fire extinguishing across the three fire stations, and no differences in the levels in post-shift samples without fire extinguishing between the baseline and intervention periods. ^2^ Compared to pre-shift levels, ^3^ Compared to post-shift levels without fire, ^4^ Compared to post-shift levels with fire call without intervention.

**Table 5:** Percentage change in urinary excretion levels of PAH metabolites (95% CI) using statistical Model 2 ^1^.

Intervention	∑OHNAP	OHFLU	∑OHPHE	OHPYR
Intervention (Sauna) ^2^	**−41.7 (−61.3; −12.2)**	**−33.0 (−50.3; −8.6)**	**−35.0 (−52.3; −11.3)**	**−37.5 (−60.2; −2.0)**
Intervention (Fire suit) ^2^	17.4 (−20.6; 73.3)	−2.0 (−30.9; 37.7)	−17.3 (−41.7; 18.5)	−29.5 (−62.5; 32.3)
Intervention (Shower) ^2^	−9.5 (−41.1; 39.1)	29.7 (−10.4; 89.7)	20.9 (−17.3; 76.8)	8.33 (−41.1; 99.4)
Awareness	∑OHNAP	OHFLU	∑OHPHE	OHPYR
Station 1 ^3^	24.6 (−4.9; 61.6)	**28.4 (6.2; 56.8)**	16.2 (−4.9; 41.9)	16.2 (−13.1; 55.3)
Station 2 ^3^	−9.5 (−34.3; 25.99)	−16.5 (−37.5; 11.6)	−8.6 (−32.3; 22.1)	−18.1 (−51.8; 37.7)
Station 3 ^3^	9.4 (−9.5; 32.3)	5.1 (−10.4; 24.6)	−5.8 (−19.7; 11.6)	**−25.2 (−42.9; −2.0)**

Numbers in bold indicate statistical significance. ^1^ Model 2 allows for different pre-shift levels between the baseline and intervention periods and awareness, defined as the difference in post-shift levels between the baseline and intervention periods (without fire), ^2^ Comparison of post-shift levels for baseline and intervention period (with fire call), ^3^ Comparison of post-shift levels for baseline and intervention period (without fire calls).

**Table 6:** Percentage change in dermal PAH levels (95% CI) using statistical Model 1 ^1^.

Factor	∑PAH
Post-shift w/o fire ^2^	3.0 (−30.9; 53.7)
Before shower (after fire) ^2^	**232.0 (113.8; 415.5)**
After shower (after fire) ^3^	**75.1 (6.2; 185.8)**
Intervention (Sauna) ^4^	−52.3 (7–8.6;6.2)
Intervention (Fire suit) ^5^	39.1 (−60.1; 380.7)
Intervention (Shower) ^6^	−62.5 (−86.2; 1.0)

Numbers in bold indicate statistical significance. ^1^ In Model 1, we assumed the following: no differences in the levels of pre-shift samples between the baseline and intervention periods, no variation in the effect of fire extinguishing across the three fire stations, and no differences in the levels in post-shift samples (without fire extinguishing) between the baseline and intervention periods, ^2^ Compared to pre-shift levels, ^3^ Compared to post-shift levels without fire, ^4^ Compared to post-shift levels when firefighting has occurred and sauna used afterwards, ^5^ Compared to before shower levels when fighting has occurred with use of fire suit with improved particle barrier, ^6^ Percent change to post-shift levels when firefighting has occurred and extra shower used afterwards.

**Table 7:** Percentage change in dermal PAH levels (95% CI) using statistical Model 2 ^1^.

Intervention	∑PAH
Intervention (Sauna) ^2^	−67.7 (−91.0; 16.2)
Intervention (Fire suit) ^3^	**−96.2 (−99.5; −73.8)**
Intervention (Shower) ^2^	41.9 (−57.3; 375.9)
Awareness	∑PAH
Station 1 ^4^	12.7 (−65.7; 266.9)
Station 2 ^4^	−63.6 (−90.5; 40.5)
Station 3 ^4^	−34.3 (−75.1; 71.6)

Numbers in bold indicate statistical significance. ^1^ The model allows for different pre-shift levels between the baseline and intervention periods, and awareness, defined as the difference in post-shift levels between the baseline and intervention periods. ^2^ Comparison of post-shift levels for base line and intervention period (with fire call). ^3^ Compared to before shower levels when fighting has occurred with use of fire suit with improved particle barrier. ^4^ Comparison of post-shift levels for baseline and intervention period (without fire calls).

**Table 8:** Percentage change in dermal PAH levels (95% CI) after shower using statistical Model 3.

Shower	∑PAH
After shower (baseline) ^1^	**−63.9 (−78.3; −39.5)**
After shower (intervention) ^2^	−2.9 (−44.0; 68.2)

^1^ Compared to before shower in the baseline period. ^2^ Compared to baseline after shower.

## Data Availability

The data presented in this study are available in aggregated form on reasonable request from the corresponding author due to ethical restrictions.

## Supplementary Materials

The following supporting information can be downloaded at: https://www.mdpi.com/article/10.3390/jox15050150/s1, Figure S1: Illustration of dermal wipe sampling adjacent areas at the different time points; Figure S2: Illustration of flow path with the valves in position 2; Figure S3a: Urinary levels of creatinine-adjusted hydroxy naphthalenes (∑OHNAP (1 + 2-OHNAP)) in participants at baseline and intervention period across work shifts with and without fire calls; Figure S3b: Urinary levels of creatinine-adjusted 2-hydroxyflurene (2-OHFLU) in participants at baseline and intervention period across work shifts with and without fire calls; Figure S3c: Urinary levels of creatinine-adjusted ∑OHPHE (2 + 3 + 4 OHPHE) in participants at baseline and intervention period across work shifts with and without fire calls; Table S1: Solvent gradient of the binary and quaternary pumps and position of the column switch during the run; Table S2: Valve positions and resulting flow path; Table S3: Retention time (RT), collision energy (CE), and MRM transitions for the individual PAHs; Table S4: Median levels (P5, P95) of urinary PAH metabolites pre- and post-shift for the three different fire stations stratified by No fire call (baseline and intervention period combined), fire call in the baseline period, and fire call in the intervention period; Table S5a: Median levels (P5, P95) of dermal PAH levels pre- and post-shift for Fire Station 1 stratified by No fire call (baseline and intervention period combined), fire call in the baseline period, and fire call in the intervention period; Table S5b: Median levels (P5, P95) of dermal PAH levels pre- and post-shift for Fire Station 2 stratified by No fire call (baseline and intervention period combined), fire call in the baseline period, and fire call in the intervention period; Table S5c: Median levels (P5, P95) of dermal PAH levels pre- and post-shift for Fire Station 3 stratified by No fire call (baseline and intervention period combined), fire call in the baseline period, and fire call in the intervention period.

## Abbreviations

The following abbreviations are used in this manuscript:

OH-PAHs	hydroxylated PAHs
1-OHPYR	1-hydroxypyrene
1-OHNAP	1-hydroxynaphthalene
2-OHNAP	2-hydroxynaphthalene
2-OHFLU	2-hydroxyfluorene
1-OHPHE	1-hydroxyphenanthrene
2 + 3-OHPHE	2 and 3-hydroxyphenanthrene
4-OHPHE	4-hydroxyphenanthrene
BMI	Body Mass Index
GC–MS/MS	Gas Chromatography with tandem Mass Spectrometry
LOQ	Limit of Quantification
PAH	Polycyclic Aromatic Hydrocarbons
