# Supplementary material for: Effects of Different Interventions Aimed at Reducing Dermal and Internal Polycyclic Aromatic Hydrocarbon Exposure Among Firefighters
Source: J Xenobiot. 2025 Sep 16;15(5):150. doi: 10.3390/jox15050150 (PMC12452719; doi:10.3390/jox15050150)
Supplement: Supplementary file 1 [file jox-15-00150-s001.zip › Figure S1_JoX.pdf]

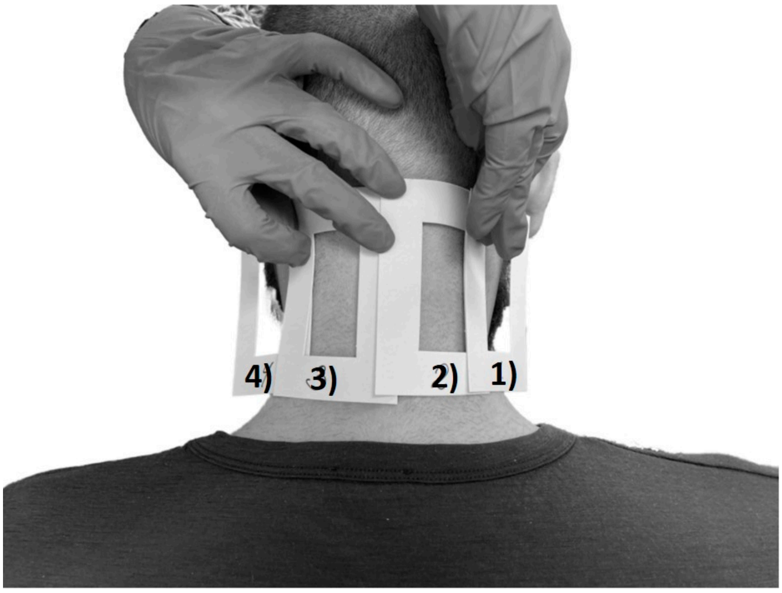

Figure S1. Illustration of dermal wipe sampling adjacent areas at the different time points: 1) pre-shift; 2) before shower; 3) after shower; and 4) post-shift
