# Supplementary material for: Effects of Different Interventions Aimed at Reducing Dermal and Internal Polycyclic Aromatic Hydrocarbon Exposure Among Firefighters
Source: J Xenobiot. 2025 Sep 16;15(5):150. doi: 10.3390/jox15050150 (PMC12452719; doi:10.3390/jox15050150)
Supplement: Supplementary file 1 [file jox-15-00150-s001.zip › Figure S3a_JoX.pdf]

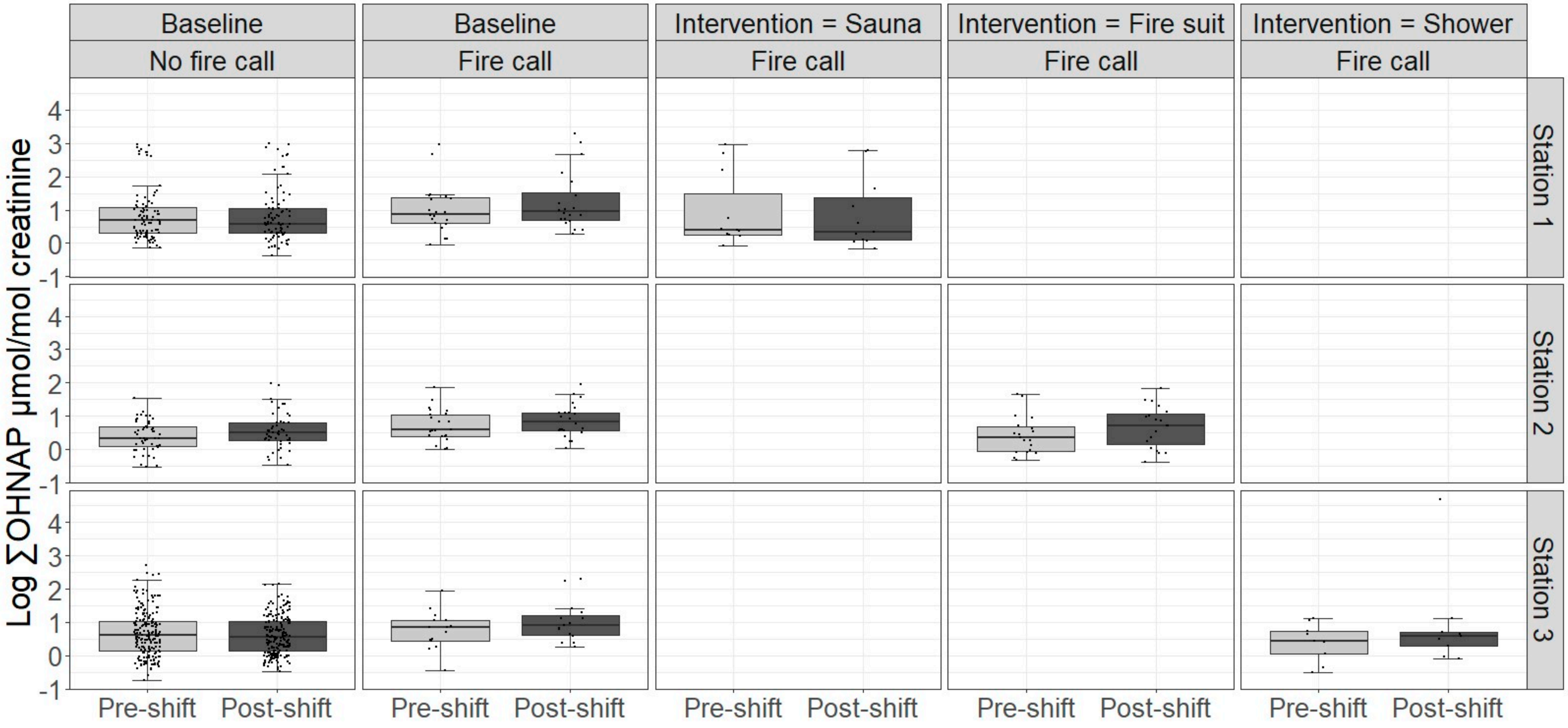

Figure S3a. Urinary levels of creatinine adjusted hydroxy naphthalenes (∑OHNAP (1+2-OHNAP)) in participants at baseline and intervention period across work shifts with and without fire calls. Boxplots represent the median and interquartile range; whiskers extend to 1.5 times the interquartile range. Points represent individual results.
