# Supplementary material for: Effects of Different Interventions Aimed at Reducing Dermal and Internal Polycyclic Aromatic Hydrocarbon Exposure Among Firefighters
Source: J Xenobiot. 2025 Sep 16;15(5):150. doi: 10.3390/jox15050150 (PMC12452719; doi:10.3390/jox15050150)
Supplement: Supplementary file 1 [file jox-15-00150-s001.zip › Table S1_JoX.pdf]

Table S1. Solvent gradient of the binary and quaternary pumps and position of the column switch during the run.

	**Binary pump** (A: water / B: MeOH)			**Quaternary pump** (A: water / B: MeOH)			**Column switches**	
**Step**	**Time**	% B (MeOH)	Flow (mL/min)	Time	% B (MeOH)	Flow (mL/min)	Time	Position
Load & Wash	**0.0**	10	1	0	35	0.35	0	1
	**2.0**	10	1	3	35	0.35		
	**2.05**	20	1					
	**4.0**	20	1	3.1	35	0		
Elution	**4.05**	35	0.35				4.05	2
	**26**	75	0.35					
Column wash	**26.05**	95	0.35	25.9	35	0.35	26	3
	**26.1**	95	1.5	26.00	95	0.35		
	**29.0**	95	1.5	29.0	95	0.35		
Equilibration	**29.1**	10	1	29.1	35	0.35		
	**31.0**	10	1	31.0	35	0.35		

The analyses PAH metabolites in urine followed the method described in Frederiksen et al. with minor modifications. Most importantly, the analyses were performed on different LC-system i.e. Agilent Infinity II 1290 LC system consististing of a G7167B Multisampler, G4227A FlexCube, G7116B column oven, G7120A Binary pump and a G7104A Quaternary pump coupled to a 6495C triple quadrupole MS. The system was equipped with two 10-port and a 6-port switching valve allowing separate wash of the SPE-column and analytical column and the capability of backflushing both columns, reducing column backpressure build up over time, and increasing the lifetime of both columns. The updated elution program is given in Table S1.
