# Supplementary material for: Effects of Different Interventions Aimed at Reducing Dermal and Internal Polycyclic Aromatic Hydrocarbon Exposure Among Firefighters
Source: J Xenobiot. 2025 Sep 16;15(5):150. doi: 10.3390/jox15050150 (PMC12452719; doi:10.3390/jox15050150)
Supplement: Supplementary file 1 [file jox-15-00150-s001.zip › Table S2_JoX.pdf]

Table S2. Valve positions and resulting flow path.

Position	Flow Path
1	Binary pump -> SPE column -> Waste and Quaternary pump -> C18 column -> Waste
2	Binary pump -> SPE column -> C18 column -> MS and Quaternary pump -> Waste
3	Binary pump -> SPE column backflush -> Waste and Quaternary pump -> C18 backflush -> Waste
