# Supplementary material for: Effects of Different Interventions Aimed at Reducing Dermal and Internal Polycyclic Aromatic Hydrocarbon Exposure Among Firefighters
Source: J Xenobiot. 2025 Sep 16;15(5):150. doi: 10.3390/jox15050150 (PMC12452719; doi:10.3390/jox15050150)
Supplement: Supplementary file 1 [file jox-15-00150-s001.zip › Table S3_JoX.pdf]

Table S3. Retention time (RT), collision energy (CE) and MRM transitions for the individual PAHs.

PAH	RT	CE	MRM transition
Naphthalene d8	9.17	20	136 → 108
Naphthalene	9.25	20	128 → 102
Acenaphthylene d8	13.25	20	160 → 158
Acenaphthylene	13.27	20	152 → 151
Acenapthtene d10	13.56	20	164 → 162
Acenaphthene	13.70	20	154 → 153
Fluorene d10	14.91	20	176 → 174
Fluorene	14.96	20	166 → 165
Phenanthrene d10	17.49	20	188 → 160
Phenanthrene	17.57	20	178 → 176
Anthracene d10	17.68	20	188 → 184
Anthracene	17.70	20	178 → 178
Fluoranthene d10	20.81	20	212 → 208
Fluoranthene	20.89	20	202 → 201
Pyrene d10	21.43	20	212 → 208
Pyrene	21.49	20	202 → 201
Benz(a)anthracene d12	25.75	20	240 → 236
Benz(a)anthracene	25.87	20	228 → 226
Chrysene d12	25.96	20	240 → 236
Chrysene	25.12	20	228 → 227
Benzo(k)fluoranthene d12	33.94	20	264 → 260
Benzo(b)fluoranthene d12	33.94	20	264 → 260
Benzo(k)fluoranthene	34.13	20	252 → 250
Benzo(b)fluoranthene	34.13	20	252 → 250
Benzo(a)pyrene d12	37.23	25	264 → 260
Benzo(a)pyrene	37.53	25	252 → 250
Dibenz(ah)anthrancene D14	47.46	35	292 → 288
Dibenz(ah)anthrancene	47.73	35	278 → 276
Ideno(123cd)pyrene D12	47.96	35	288 → 284
Ideno(123cd)pyrene	48.14	35	276 → 274
Benzo(ghi)perylene D12	50.43	35	288 → 284
Benzo(ghi)perylene	50.71	35	276 → 274
