# Supplementary material for: Effects of Different Interventions Aimed at Reducing Dermal and Internal Polycyclic Aromatic Hydrocarbon Exposure Among Firefighters
Source: J Xenobiot. 2025 Sep 16;15(5):150. doi: 10.3390/jox15050150 (PMC12452719; doi:10.3390/jox15050150)
Supplement: Supplementary file 1 [file jox-15-00150-s001.zip › Table S4_JoX.pdf]

Table S4. Median levels (P5, P95) of urinary PAH metabolites pre- and post-shift for the three different fire stations stratified by No fire call (baseline and intervention period combined), Fire call in the baseline period and Fire call in the intervention period.

	**Station 1 (Intervention = Sauna)**					
**Conc unit µmol/mol Creatinine**	No fire		Fire: Baseline		Fire: Intervention	
**Metabolite**	Pre-shift	Post-shift	Pre-shift	Post-shift	Pre-shift	Post-shift
**∑Hydroxy naphthalene**	1.98 (1.02; 15.55)	1.80 (0.91; 14.33)	2.39 (1.12; 14.88)	2.58 (1.47; 21.18)	1.47 (1.07; 17.43)	1.39 (0.94; 16.03)
**2-Hydroxy fluorene**	0.083 (0.037; 0.98)	0.082 (0.045; 0.70)	0.063 (0.039; 1.04)	0.072 (0.044; 1.20)	0.091 (0.046; 0.87)	0.075 (0.060; 0.83)
**∑Hydroxy phenanthrene**	0.15 (0.072; 0.36)	0.14 (0.083; 0.35)	0.15 (0.079; 0.37)	0.18 (0.094; 0.68)	0.18 (0.13; 0.31)	0.16 (0.12; 0.32)
**1-Hydroxy pyrene**	0.021 (0.0089; 0.099)	0.028 (0.013; 0.090)	0.020 (0.0091;0.099)	0.052 (0.015; 0.19)	0.027 (0.018; 0.12)	0.037 (0.024; 0.13)
**Sum**	2.17 (1.21; 17.12)	2.03 (1.17; 15.30)	2.58 (1.41; 16.39)	2.81 (1.69; 23.08)	1.79 (1.33; 18.69)	1.62 (1.19; 17.31)
**N (samples)**	87	89	20	20	10	11
**N (firefighters)**	11	11	7	7	5	5
	**Station 2 (Intervention = Fire suit)**					
**Conc unit µmol/mol Creatinine**	No fire		Fire: Baseline		Fire: Intervention	
**Metabolite**	Pre-shift	Post-shift	Pre-shift	Post-shift	Pre-shift	Post-shift
**∑Hydroxy naphthalene**	1.38 (0.69; 2.83)	1.67 (0.78; 4.54)	1.80 (1.05; 4.33)	2.33 (1.28; 5.15)	1.41 (0.76; 4.96)	2.06 (0.87; 4.53)
**2-Hydroxy fluorene**	0.085 (0.031; 0.17)	0.12 (0.056; 0.30)	0.11 (0.074; 0.17)	0.16 (0.10; 0.30)	0.086 (0.045; 0.19)	0.14 (0.048; 0.29)
**∑Hydroxy phenanthrene**	0.13 (0.039; 0.24)	0.22 (0.12; 0.50)	0.19 (0.12; 0.33)	0.31 (0.21; 0.73)	0.17 (0.065; 0.26)	0.22 (0.097; 0.51)
**1-Hydroxy pyrene**	0.023 (0.011; 0.074)	0.037 (0.018; 0.28)	0.034 (0.020; 0.13)	0.088 (0.046; 0.41)	0.030 (0.011; 0.054)	0.053 (0.013; 0.13)
**Sum**	1.73 (0.78; 3.15)	2.10 (1.06; 5.55)	2.15 (1.33; 4.76)	3.03 (1.62; 6.36)	1.77 (0.98; 5.63)	2.52 (1.05; 5.33)
**N (samples)**	49	50	20	20	19	19
**N (firefighters)**	8	8	8	8	7	7
	**Station 3 (Intervention = Extra shower)**					
**Conc unit µmol/mol Creatinine**	No fire		Fire: Baseline		Fire: Intervention	
**Metabolite**	Pre-shift	Post-shift	Pre-shift	Post-shift	Pre-shift	Post-shift
**∑Hydroxy naphthalene**	1.83 (0.76; 6.68)	1.73 (0.81; 4.97)	2.31 (1.04; 5.02)	2.49 (1.41; 9.47)	1.56 (0.64; 2.97)	1.81 (0.94; 66.83)
**2-Hydroxy fluorene**	0.085 (0.031; 0.26)	0.080 (0.039; 0.26)	0.059 (0.038; 0.24)	0.14 (0.047; 0.36)	0.069 (0.030; 0.12)	0.083 (0.060; 1.39)
**∑Hydroxy phenanthrene**	0.15 (0.066; 0.67)	0.15 (0.077; 0.52)	0.11 (0.066; 0.60)	0.18 (0.082; 0.66)	0.13 (0.065; 0.57)	0.15 (0.10; 1.74)
**1-Hydroxy pyrene**	0.021 (0.00; 0.10)	0.025 (0.012; 0.11)	0.025 (0.012; 0.097)	0.065 (0.027; 0.15)	0.019 (0.013; 0.029)	0.030 (0.021;0.20)
**Sum**	2.21 (1.07; 7.10)	2.08 (1.13; 5.34)	2.90 (1.15; 5.57)	2.85 (1.57; 10.38)	2.10 (0.91; 3.23)	2.06 (1.31; 70.06)
**N (samples)**	194	196	15	15	9	9
**N (firefighters)**	7	7	5	5	6	6
