# Supplementary material for: Effects of Different Interventions Aimed at Reducing Dermal and Internal Polycyclic Aromatic Hydrocarbon Exposure Among Firefighters
Source: J Xenobiot. 2025 Sep 16;15(5):150. doi: 10.3390/jox15050150 (PMC12452719; doi:10.3390/jox15050150)
Supplement: Supplementary file 1 [file jox-15-00150-s001.zip › Table S5_a_JoX.pdf]

Table S5.a Median levels (P5, P95) of dermal PAH levels pre- and post-shift for fire station 1 stratified by No fire call (baseline and intervention period combined), Fire call in the baseline period and Fire call in the intervention period.

**Unit: ng/cm²**	**Station 1 (Intervention = Sauna)**									
	No fire		Fire no intervention				Fire with intervention			
**PAH**	Pre-shift	Post-shift	Pre-shift	Before shower	After shower	Post-shift	Pre-shift	Before shower	After shower	Post-shift
**Naphthalene**	0 (0; 0)	0 (0; 0)	0 (0; 0)	0 (0; 8.19)	0 (0; 3.62)	-	0 (0; 0.13)	0.049 (0; 0.18)	0 (0; 0.14)	0.015 (0; 0.055)
**Acenapthylene**	0 (0; 0.034)	0 (0; 0.032)	0.002 (0; 0.034)	0.004 (0; 0.086)	0 (0; 0.025)	-	0 (0; 0)	0 (0; 0)	0 (0; 0)	0 (0; 0)
**Acenaphtene**	0 (0; 0.034)	0 (0; 0.035)	0.008 (0; 0.042)	0 (0; 0.033)	0 (0; 0.031)	-	0 (0; 0)	0 (0; 0)	0 (0; 0)	0 (0; 0)
**Fluorene**	0 (0; 0.68)	0 (0; 0.16)	0 (0; 0.25)	0.12 (0; 0.98)	0 (0; 0.96)	-	0 (0; 0.021)	0 (0; 0.059)	0 (0; 0.051)	0 (0; 0)
**Phenanthrene**	0.045 (0.001; 0.51)	0.035 (0; 0.73)	0.031 (0.004; 0.066)	0.15 (0; 1.20)	0.036 (0; 0.48)	-	0.22 (0.061; 0.40)	0.36 (0; 0.67)	0 (0; 0.30)	0.15 (0; 0.22)
**Anthracene**	0.014 (0; 0.033)	0.013 (0; 0.055)	0.016 (0; 0.041)	0.016 (0; 0.21)	0.009 (0; 0.026)	-	0 (0; 0)	0 (0; 0)	0 (0; 0)	0 (0; 0)
**Fluoranthene**	0.034 (0; 0.15)	0.008 (0; 0.086)	0.031 (0.007; 0.096)	0.056 (0; 0.96)	0.025 (0; 0.36)	-	0 (0; 0)	0.13 (0; 0.47)	0 (0; 0.15)	0 (0; 0)
**Pyrene**	0.018 (0; 0.31)	0.013 (0; 0.060)	0.028 (0;0.12)	0.095 (0; 1.45)	0.025 (0; 0.46)	-	0 (0; 0.21)	0.36 (0; 0.69)	0 (0; 0.22)	0.003 (0; 0.089)
**Benzo(a)anthracene**	0 (0; 0)	0 (0; 0)	0 (0; 0.015)	0 (0; 0.055)	0 (0; 0.067)	-	0 (0; 0)	0 (0; 0)	0 (0; 0)	0 (0; 0)
**Chrysene**	0 (0; 0.082)	0 (0; 0.060)	0.016 (0; 0.10)	0.004 (0; 0.087)	0.002 (0; 0.12)	-	0 (0; 0)	0 (0; 0)	0 (0; 0)	0 (0; 0)
**Benzo(k+b)fluoranthene[1]**	0 (0; 0.41)	0 (0; 0.19)	0.040 (0; 0.61)	0 (0; 1.12)	0.005 (0; 0.55	-	0 (0; 1.08)	0.58 (0; 4.00)	0 (0; 1.95)	0.016 (0; 1.63)
**Benzo(a)pyrene**	0 (0; 0)	0 (0; 0)	0 (0; 0)	0 (0; 0.32)	0 (0; 0.58)	-	0 (0; 0.90)	0 (0; 2.68)	0 (0; 0)	0 (0; 0)
**∑PAH neck[2]**	0.27 (0.055; 1.14)	0.14 (0.065; 0.79)	0.37 (0.036; 1.10)	1.07 (0.13; 9.24)	0.63 (0.057; 5.01)	-	0.44 (0.18; 2.18)	1.46 (0.52; 7.91)	0.47 (0.005; 1.98)	0.19 (0.11; 1.88)
**N (samples)**	28	27	10	20	18	1	8	15	13	6
**N (firefighters with measurements)**	10	10	6	7	7	1	4	6	6	4

[1]Complete separation was not possible for benzo[b]fluoranthene and benzo[k]fluoranthene, and therefore they were reported as the sum (benzo[k+b]fluoranthene). [2]Due to instable and occasionally high blank levels, the levels of dibenz(ah)anthrancene, ideno(123cd)pyrene and benzo(ghi)perylene should be interpreted with caution and therefore they were not included in the statistical analysis.
