# Supplementary material for: Effects of Different Interventions Aimed at Reducing Dermal and Internal Polycyclic Aromatic Hydrocarbon Exposure Among Firefighters
Source: J Xenobiot. 2025 Sep 16;15(5):150. doi: 10.3390/jox15050150 (PMC12452719; doi:10.3390/jox15050150)
Supplement: Supplementary file 1 [file jox-15-00150-s001.zip › Table S5_b_JoX.pdf]

Table S5.b Median levels (P5, P95) of dermal PAH levels pre- and post-shift for fire station 2 stratified by No fire call (baseline and intervention period combined), Fire call in the baseline period and Fire call in the intervention period.

	Station 2 (Intervention = Fire suit)									
**Unit: ng/cm²**	No fire		Fire no intervention				Fire with intervention			
**PAH**	Pre-shift	Post-shift	Pre-shift	Before shower	After shower	Post-shift	Pre-shift	Before shower	After shower	Post-shift
**Naphthalene**	0 (0; 0)	0 (0; 0)	0 (0; 0)	0 (0; 0.026)	0 (0; 0)	-	0 (0; 0.77)	0 (0; 0)	0 (0; 0)	0 (0; 0)
**Acenapthylene**	0 (0; 0.022)	0 (0; 0.027)	0 (0; 0.019)	0.016 (0; 0.47)	0 (0; 0)	-	0 (0; 0.045)	0 (0; 0.031)	0 (0; 0.040)	0 (0; 0.11)
**Acenaphtene**	0 (0; 0.027)	0 (0; 0.023)	0 (0; 0)	0 (0; 0)	0 (0; 0)	-	0 (0; 0)	0 (0; 0)	0 (0; 0)	0 (0; 0)
**Fluorene**	0 (0; 0.061)	0 (0; 0.26)	0 (0; 0.30)	0 (0; 0)	0 (0; 0.047)	-	0 (0; 0.053)	0 (0; 0.037)	0 (0; 0.027)	0 (0; 0.019)
**Phenanthrene**	0.013 (0; 0.24)	0.081 (0; 0.23)	0 (0; 0.53)	0.36 (0.022; 2.24)	0.081 (0; 0.29)	-	0.028 (0; 0.14)	0.14 (0; 2.24)	0.060 (0; 0.19)	0.032 (0; 0.14)
**Anthracene**	0 (0; 0.043)	0 (0; 0.013)	0 (0; 0.28)	0 (0; 0)	0 (0; 0)	-	0(0; 0.17)	0 (0; 0)	0 (0; 0)	0 (0; 0)
**Fluoranthene**	0 (0; 0.088)	0.018 (0; 0.077)	0.003 (0; 0.13)	0.28 (0.053; 1.27)	0.021 (0; 0.36)	-	0.31 (0; 0.88)	0.38 (0; 0.87)	0.51 (0.066; 0.57)	0.23 (0; 2.91)
**Pyrene**	0 (0; 0)	0 (0; 0)	0 (0; 0.045)	0.34 (0; 1.01)	0.069 (0; 0.33)	-	0 (0; 0.032)	0 (0; 0.23)	0 (0; 0.071)	0 (0; 0.003)
**Benzo(a)anthracene**	0 (0; 0.0003)	0 (0; 0.002)	0 (0; 0)	0 (0; 0)	0 (0; 0.002)	-	0 (0; 0.062)	0 (0; 0)	0 (0; 0)	0 (0; 0.31)
**Chrysene**	0 (0; 0)	0 (0; 0.002)	0 (0; 0.016)	0 (0; 0.15)	0 (0; 0)	-	0 (0; 0.11)	0 (0; 0)	0 (0; 0)	0 (0; 0.83)
**Benzo(b+k)fluoranthene[1]**	0 (0; 0)	0 (0; 0)	0 (0; 0)	0 (0; 0)	0 (0; 0)	-	0.25 (0; 3.81)	0 (0; 3.32)	0 (0; 4.59)	0.10 (0; 3.13)
**Benzo(a)pyrene**	0 (0; 0)	0 (0; 0)	0 (0; 0)	0 (0; 0)	0 (0; 0)	-	0 (0; 2.07)	0 (0; 3.46)	0 (0; 0.29)	0 (0; 2.86)
**∑PAH neck[2]**	0.063 (0; 0.37)	0.11 (0.040; 0.44)	0.20 (0; 0.71)	1.02 (0.15; 4.93)	0.15 (0; 0.97)	-	0.90 (0.17; 6.62)	1.96 (0.18; 7.94)	0.68 (0.49; 5.19)	0.66 (0.15; 9.82)
**N (samples)**	18	15	8	9	7	0	10	7	6	12
**N (firefighters with measurements)**	7	7	5	5	4	0	6	4	4	6

[1]Complete separation was not possible for benzo[b]fluoranthene and benzo[k]fluoranthene, and therefore they were reported as the sum (benzo[k+b]fluoranthene). [2]Due to instable and occasionally high blank levels, the levels of dibenz(ah)anthrancene, ideno(123cd)pyrene and benzo(ghi)perylene should be interpreted with caution and therefore they were not included in the statistical analysis.
