# Supplementary material for: Effects of Different Interventions Aimed at Reducing Dermal and Internal Polycyclic Aromatic Hydrocarbon Exposure Among Firefighters
Source: J Xenobiot. 2025 Sep 16;15(5):150. doi: 10.3390/jox15050150 (PMC12452719; doi:10.3390/jox15050150)
Supplement: Supplementary file 1 [file jox-15-00150-s001.zip › Table S5_c_JoX.pdf]

Table S5.c Median levels (P5, P95) of dermal PAH levels pre- and post-shift for fire station 3 stratified by No fire call (baseline and intervention period combined), Fire call in the baseline period and Fire call in the intervention period.

	Station 3 (Intervention = Shower)									
**Unit: ng/cm²**	No fire		Fire no intervention				Fire with intervention			
**PAH**	Pre-shift	Post-shift	Pre-shift	Before shower	After shower	Post-shift	Pre-shift	Before shower	After shower	Post-shift
**Naphthalene**	0 (0; 0.012)	0 (0; 0.040)	0 (0; 0.070)	0 (0; 0.047)	0 (0; 0.013)	-	0.081 (0; 0.17)	0.093 (0; 0.28)	0.062 (0; 0.11)	-
**Acenapthylene**	0 (0; 0.080)	0 (0; 0.018)	0.008 (0; 0.031)	0.004 (0; 0.026)	0.005 (0; 0.016)	-	0.012 (0.001; 0.025)	0.019 (0.001; 0.39)	0.016 (0; 0.061)	-
**Acenaphtene**	0 (0; 0.044)	0 (0; 0.038)	0.017 (0; 0.029)	0.010 (0; 0.054)	0.016 (0; 0.036)	-	0 (0; 0)	0 (0; 0)	0 (0; 0)	-
**Fluorene**	0 (0; 0.016)	0 (0; 0.15)	0 (0; 0.047)	0 (0; 0.078)	0 (0; 0.041)	-	0.033 (0; 0.055)	0.049 (0; 0.34)	0.014 (0.004; 0.11)	-
**Phenanthrene**	0 (0; 1.61)	0 (0; 0.43)	0 (0; 0.11)	0.2 (0; 0.53)	0 (0; 0.082)	-	0.008 (0; 0.98)	0.11 (0; 3.22)	0.03 (0; 0.72)	-
**Anthracene**	0.015 (0; 0.16)	0.0028 (0; 0.13)	0.013 (0; 0.31)	0.014 (0; 0.25)	0.005 (0; 0.15)	-	0 (0; 0)	0 (0; 0.55)	0 (0: 0.13)	-
**Fluoranthene**	0 (0; 0.57)	0 (0; 0.17)	0 (0; 0.41)	0.12 (0; 0.29)	0.012 (0; 0.17)	-	0.021 (0; 0.11)	0.066 (0.021; 1.89)	0.092 (0.003; 0.41)	-
**Pyrene**	0 (0; 0.32)	0 (0; 0.26)	0 (0; 0.21)	0.07 (0; 0.37)	0 (0; 0.065)	-	0.051 (0.004; 0.22)	0.086 (0.044; 1.96)	0.070 (0.003; 0.53)	-
**Benzo(a)anthracene**	0.15 (0; 1.04)	0.11 (0; 0.73)	0.17 (0; 0.54)	0.11 (0; 0.38)	0.076 (0; 0.28)	-	0 (0; 0)	0 (0; 0.33)	0 (0; 0)	-
**Chrysene**	0.22 (0; 1.56)	0.21 (0; 1.20)	0.063 (0; 0.65)	0.061 (0; 0.37)	0.16 (0; 0.43)	-	0 (0; 0.11)	0 (0; 0.55)	0 (0; 0.18)	-
**Benzo(k+b)fluoranthene**[1]	0 (0; 0.98)	0 (0; 0.68)	0 (0; 1.17)	0 (0; 0.90)	0 (0; 0.85)	-	0 (0;0)	0 (0; 0)	0 (0; 0.43)	-
**Benzo(a)pyrene**	0 (0; 0.34)	0 (0; 0.27)	0 (0; 0)	0 (0; 0.35)	0 (0; 0.12)	-	0 (0; 1.05)	0 (0; 0.73)	0 (0; 0.49)	-
**∑PAH neck**[2]	1.42 (0.24; 4.14)	0.98 (0.13; 3.32)	0.69 (0.078; 2.55)	0.91 (0.25; 2.36)	0.53 (0.14; 1.34)	-	0.16 (0.063; 2.57)	0.51 (0.20; 10.20)	0.66 (0.053; 2.10)	-
**N (samples)**	23	21	14	14	13	2	8	13	9	3
**N (firefighters with measurements)**	7	7	5	5	5	2	6	7	6	3

[1]Complete separation was not possible for benzo[b]fluoranthene and benzo[k]fluoranthene, and therefore they were reported as the sum (benzo[k+b]fluoranthene). [2]Due to instable and occasionally high blank levels, the levels of dibenz(ah)anthrancene, ideno(123cd)pyrene and benzo(ghi)perylene should be interpreted with caution and therefore they were not included in the statistical analysis.
