# Supplementary figures and images for: Effects of Different Interventions Aimed at Reducing Dermal and Internal Polycyclic Aromatic Hydrocarbon Exposure Among Firefighters
Source: J Xenobiot. 2025 Sep 16;15(5):150. doi: 10.3390/jox15050150 (PMC12452719; doi:10.3390/jox15050150)

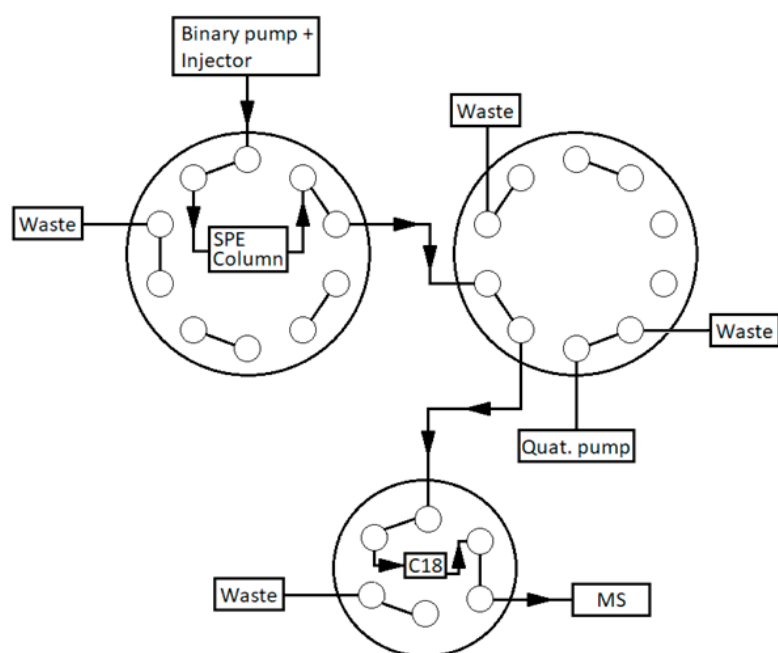

**Figure S2.** Illustration of flow path with the valves in position 2.

Supplement: Supplementary file 1 [file jox-15-00150-s001.zip › Figure S2_JoX.pdf]
